# Interface Coassembly and Polymerization on Magnetic Colloids: Toward Core–Shell Functional Mesoporous Polymer Microspheres and Their Carbon Derivatives

**DOI:** 10.1002/advs.202000443

**Published:** 2020-04-30

**Authors:** Panpan Pan, Tong Zhang, Qin Yue, Ahmed A. Elzatahry, Abdulaziz Alghamdi, Xiaowei Cheng, Yonghui Deng

**Affiliations:** ^1^ Department of Chemistry Department of Chemistry, Department of Gastroenterology and Hepatology, Zhongshan Hospital, State Key Laboratory of Molecular Engineering of Polymers Fudan University Shanghai 200433 China; ^2^ Institute of Fundamental and Frontier Sciences University of Electronic Science and Technology of China Chengdu 610051 China; ^3^ Materials Science and Technology Program, College of Arts and Sciences Qatar University PO Box 2713 Doha Qatar; ^4^ Department of Chemistry, College of Science King Saud University PO Box 2455 Riyadh 11451 Saudi Arabia; ^5^ State Key Lab of Transducer Technology, Shanghai Institute of Microsystem and Information Technology Chinese Academy of Sciences Shanghai 200050 China

**Keywords:** core–shell, interface coassembly, magnetic materials, mesoporous carbon, mesoporous polydopamine

## Abstract

Core–shell structured magnetic mesoporous polymer or carbon‐based microspheres not only possess the combined merits of magnetic particles and stable mesoporous shell but also provide various organic functional groups for further modification and immobilization of active sites, thus opening up more possibility for various applications. Herein, a bottom‐up soft‐templating strategy is developed to controllably synthesize core–shell magnetic mesoporous polydopamine microspheres (MMP) and their derivative magnetic mesoporous carbon (MMC) microspheres via an amphiphilic block copolymer‐directed interface assembly and polymerization (denoted as abc‐DIAP) approach. The obtained uniform MMP microspheres have a well‐defined structure consisting of magnetic core, silica middle layer and mesoporous PDA shell, uniform mesopores of 11.9 nm, high specific surface areas (235.6 m^2^ g^−1^) and rich functional groups. They show fast magnetic separation speed and superior performance in selective adsorption of Cyt.C from complex biosample solutions. Moreover, they can be in situ converted into core–shell magnetic mesoporous carbon (MMC) for efficient in‐pore immobilization of ultrafine Au nanoparticles for high‐efficiency catalytic epoxidation of styrene with high conversion (88.6%) and selectivity (90.1%) toward styrene oxide.

## Graphical abstract

An interfacial coassembly and polymerization is developed for the fabrication of core–shell magnetic mesoporous polymers with ordered and accessible mesopore. The obtained well‐defined and tailorable core–shell magnetic polymers possessing abundant functional organic groups show potential applications in enzyme immobilization and size‐selective enrichment. The derived magnetic mesoporous carbon microspheres exhibit desirable carriers for loading noble metal nanoparticles and heterogeneous catalysis.

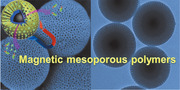

Ever since their discovery, ordered mesoporous materials have aroused tremendous research interests owing to their unique properties including tunable mesostructures, high surface area, large pore volume, and various framework components.^[^ ^1^, ^2^, ^3^ ^]^ Benefiting from these merits, mesoporous materials exhibit potential applications in catalysis,^[^ ^4^, ^5^ ^]^ adsorption and separation,^[^ ^6^, ^7^ ^]^ biomedicine,^[^ ^8^, ^9^ ^]^ energy storage and conversion,^[^ ^10^, ^11^ ^]^ and sensors.^[^ ^12^, ^13^, ^14^ ^]^ Among various strategies, the soft templating method^[^ ^15^ ^]^ through the flexible coassembly of surfactants (e.g., Pluronic F127, PEO_106_‐PPO_70_‐PEO_106_) and framework precursors via electrostatic attraction or hydrogen bond interaction can achieve precisely control syntheses of mesoporous materials with desirable pore parameters, nanostructures, compositions, and surface properties. On the other hand, magnetic particles^[^ ^16^ ^]^ possess many unique physical and chemical properties such as magnetic responsiveness, magnetocaloric effect, magnetic resonance, etc, and as a result, they show wide applications in magnetic separation,^[^ ^17^ ^]^ catalysis,^[^ ^18^ ^]^ magnetic targeting hyperthermia for cancer treatment,^[^ ^19^ ^]^ and contrast agents for disease diagnosis.^[^ ^20^ ^]^ Combining the merits of magnetic particles with mesoporous materials to fabricate core‐shell structures with an inner magnetic cores and mesoporous shell^[^ ^21^, ^22^, ^23^ ^]^ have emerged as an attractive research hotspot, because the mesoporous shell can provide high surface area for cargo delivery, storage and immobilization and inner magnetic core endows the materials quick magnetic responsiveness and magnetocaloric properties, etc.

To date, most of the reported core–shell magnetic mesoporous materials monotonically have a siliceous shell,^[^ ^24^, ^25^, ^26^, ^27^, ^28^, ^29^, ^30^, ^31^, ^32^, ^33^, ^34^, ^35^, ^36^, ^37^ ^]^ which impose limitation to their applications, especially in some harsh conditions. Core–shell magnetic materials with flexible mesoporous polymer or rigid carbon shell have seldom reported. Since polymer possesses various organic functional groups to endow the material unique surface properties and facilitate further modification and immobilization of active sites, mesoporous polymer shell can open up more possibilities for applications such as specific recognition adsorption/separation and size‐selective enrichment. Moreover, through appropriate pyrolysis treatment, polymer shell can be further converted to carbon,^[^ ^38^, ^39^, ^40^, ^41^, ^42^, ^43^ ^]^ and this carbon shell has high chemical stability, excellent conductivity, photothermal effect, good affinity to organics, which is favorable for applications in diverse disciplines including chemistry,^[^ ^44^, ^45^, ^46^ ^]^ biology,^[^ ^47^ ^]^ electronics,^[^ ^48^ ^]^ etc. However, the well‐defined core–shell magnetic mesoporous polymer/carbon materials with ordered and accessible mesopore channels through the bottom‐up soft‐templating method have seldom been reported.

Herein, for the first time, we developed an amphiphilic block copolymer‐directed interface assembly and polymerization (denoted as abc‐DIAP) approach to controllably synthesize core–shell magnetic mesoporous polydopamine microspheres with vertically aligned pore channel in the shell (MMP‐V) and their derivative magnetic mesoporous carbon microspheres (MMC‐V). The synthesis was achieved through coassembly of amphiphilic triblock PEO_106_‐PPO_70_‐PEO_106_ copolymers (Pluronic F127) and dopamine (DA) and the simultaneous oxidative polymerization of DA at the interface of presynthesized silica‐coated magnetite particles (Fe_3_O_4_@*n*SiO_2_) in a microemulsion solution. The obtained MMP‐V exhibits regular sandwich structure with magnetic core, silica middle layer and mesoporous PDA shell, large pore sizes (11.9 nm), high surface areas (235.6 m^2^ g^−1^), rich nitrogen‐containing groups, and fast magnetic separation speed. After calcination in nitrogen, the mesoporous PDA shell can be in situ converted into mesoporous carbon with well‐retained morphology and mesostructures. Due to the good affinity of PDA and the size‐exclusion effect of mesopores, the obtained MMP‐V exhibit superior performance in selective adsorption of Cyt.C from complex biosample solutions. Moreover, ultrafine gold nanoparticles can be homogeneously deposited in the pore channels of the derivated MMC‐V, and the resultant magnetic heterogeneous catalysts exhibit excellent performance in catalyzing the epoxidation of styrene with high conversion (88.6%) and selectivity (90.1%) towards styrene oxide.

The synthesis procedure for core–shell magnetic mesoporous PDA microspheres and their derivative carbon‐based microspheres are shown in **Scheme** **1**. First, highly hydrophilic colloidal magnetic (Fe_3_O_4_) particles are coated with a layer of nonporous silica (*n*SiO_2_) via a modified Stöber method. Second, F127 molecules assemble with dopamine at the interface of Fe_3_O_4_@*n*SiO_2_ microspheres in the weakly alkaline solution consisting of water, ethanol, 1, 3,5‐trimethyl benzene (TMB) and NH_4_OH. The in situ polymerization of dopamine leads to the mesostructured DA/F127 coating on Fe_3_O_4_@*n*SiO_2_, forming Fe_3_O_4_@*n*SiO_2_@PDA/F127 microspheres. After acetone extraction of F127, MMP‐V is obtained, while calcination treatment of Fe_3_O_4_@*n*SiO_2_@PDA/F127 composite in N_2_ gives rise to MMC‐V.

**Scheme 1:**
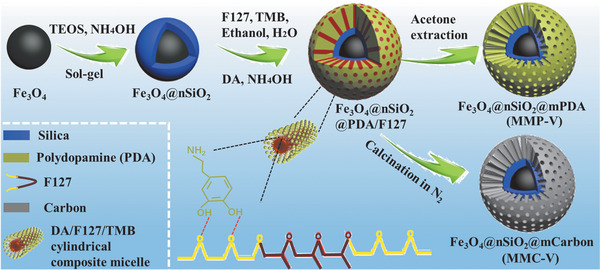
Synthesis procedures for core–shell magnetic mesoporous polydopamine (MMP) microspheres and derivated carbon (magnetic mesoporous carbon, MMC) microspheres with radial mesopore channels.

Water‐dispersible Fe_3_O_4_ particles with a uniform size of ≈100 nm (**Figure** **1**a) were synthesized via solvothermal reaction.^[^ ^49^ ^]^ After coating a layer of nonporous silica, core–shell spheres with a shell thickness of 50 nm were obtained (Figure 1b, Figure S1a, Supporting Information). This thick and dense silica shell can serve as a protective layer for iron oxide particles and provide silica‐like surface for further surface functionalization. Using F127 as a structure‐directing agent, a uniform DA/F127 composite shell can be readily deposited on Fe_3_O_4_@*n*SiO_2_ microspheres through TMB involved interface coassembly of F127 molecules and the spontaneous oxidative polymerization of dopamine under alkaline conditions. The as‐made Fe_3_O_4_@*n*SiO_2_@PDA/F127 composites have a regular spherical morphology and a mean diameter of ≈400 nm (Figure 1c, Figure S1b, Supporting Information), corresponding to an F127/PDA thickness of ≈100 nm. After removing F127, the open uniform mesopores are clearly observed to hexagonally and perpendicularly align in the surface (Figure 1d, Figure S2, Supporting Information). The mesopore size is estimated at about 12 nm (Figure 1e,f). Fourier transform infrared (FTIR) spectroscopy characterization shows (Figure S3, Supporting Information) the typical absorption peaks at 1620 and 3430 cm^−1^ are attributed to the aromatic rings and catechol —OH groups of polydopamine component,^[^ ^50^ ^]^ respectively, in the obtained MMP‐V microspheres. The disappearance of absorption peaks at 1109 cm^−1^ are ascribed to the absorption peak of C—O—C of F127 molecules, indicating complete removal of surfactants for generation of uniform mesopores. X‐ray photoelectron spectroscopy (XPS) characterization (Figure S4a–c, Supporting Information) reveals the presence of C, N, O, and Si elements. The high‐resolution profiles of C 1s show the distinct peaks of C=C (284.6 eV), C—N (285.7 eV), and O—C=O (289.1 eV) bonds. The high‐resolution profile of N 1s exhibits two main peaks at 398.3 and 399.8 eV that can be attributed to pyridinic N and pyrrolic N, respectively.

**Figure 1:**
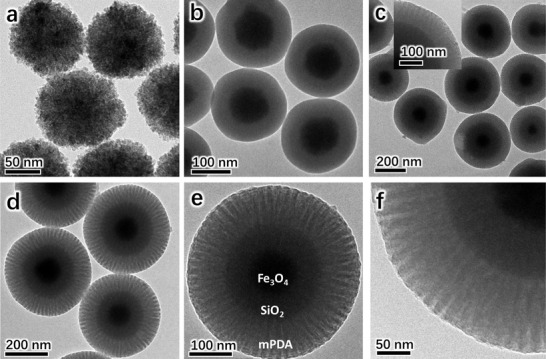
TEM images of a) as‐synthesized hydrophilic magnetite particles, b) Fe_3_O_4_@*n*SiO_2_ core–shell microspheres, c) Fe_3_O_4_@*n*SiO_2_@PDA/F127 composite microspheres obtained after interface coassembly of F127 with dopamine and spontaneous oxidative polymerization, d–f) core–shell–shell magnetic mesoporous PDA (MMP‐V) microspheres obtained after removing F127 templates via acetone extraction from the composite microspheres.

Nitrogen adsorption–desorption measurement (**Figure** **2**a) reveals that the MMP‐V microspheres have a type‐IV curve, indicating a mesoporous nanostructure. The typical H4 hysteresis loop shows two distinct desorption steps mainly due to the radially aligned trumpet‐shaped pore channels.^[^ ^51^ ^]^ The pore size distribution confirms a mean diameter of ≈11.9 nm (Figure 2b), in agreement with that estimated from TEM images. The pore size is much larger than those (about 7.0 nm) of typical pure mesoporous carbon microspheres using F127 as templates due to the pore expanding effect of TMB.^[^ ^42^, ^43^ ^]^ The BET surface area and total pore volume of MMP‐V microspheres are calculated to be 235.6 m^2^ g^−1^ and 0.16 cm^3^ g^−1^, respectively. Magnetic hysteresis loops (Figure 2d) reveal the superparamagnetic property and magnetization saturation values of 67.5 and 32.5 emu g^−1^ for Fe_3_O_4_ and MMP‐V microspheres, respectively. The superparamagnetism favors the fast separation and redispersion in the presence and absence of applied magnetic field, respectively. The obtained microspheres exhibit excellent dispersibility in polar solvents such as water and ethanol due to the presence of numerous hydrophilic groups in the PDA shell (Figure 2d, inset). To the best of our knowledge, such magnetic mesoporous polymer microspheres with well‐defined core–shell structure and highly accessible mesopores have never been reported before. As an applied magnetic field (MF) is introduced in the interface assembly process, uniform Fe_3_O_4_@*n*SiO_2_@PDA/F127 nanochains (Figure S5, Supporting Information) were generated due to MF guided directional assembly of magnetic cores.

**Figure 2:**
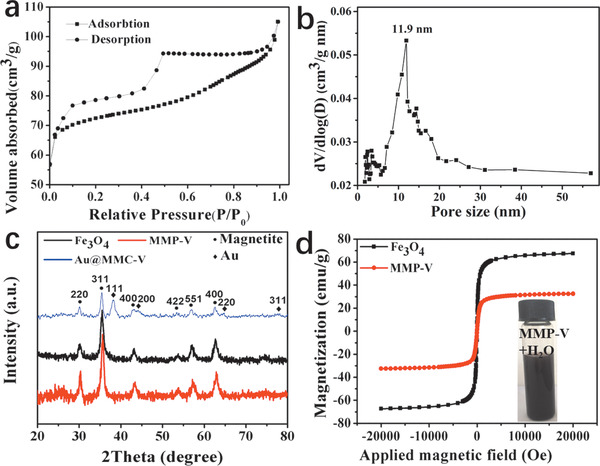
a) Nitrogen adsorption–desorption isotherms and b) pore size distribution of MMP‐V microspheres, c) XRD patterns and d) magnetic hysteresis loops of Fe_3_O_4_ particles and MMP‐V microspheres. The inset in panel d is the optical photo of MMP‐V dispersed in water.

To gain more insight into the formation of DA/F127 composite on colloidal Fe_3_O_4_@*n*SiO_2_ microspheres via this abc‐DIAP process, intermediate samples were withdrawn from the reaction solution (DA concentration: 9 mg mL^−1^). TEM observation indicates that, after reaction for 15 min, the smooth surface of Fe_3_O_4_@*n*SiO_2_ microspheres becomes rough due to the interface deposition of spherical F127/PDA composite micelles (Figure S6a,b, Supporting Information). As the reaction proceeds, PDA is generated by continuously oxidative polymerization in alkaline solution, and more DA/F127 micelles form on the surface and the isolated domains become continuous thin layers covering the magnetic seeds. On the Fe_3_O_4_@*n*SiO_2_ microsphere surface, mesostructure can be clearly observed after reaction for 20 min (Figure S6c, Supporting Information). With the continuous polymerization and interface deposition, the mesostructured F127/PDA shell grows thicker, and the spherical micelles tend to evolve into cylindrical ones^[^ ^43^ ^]^ due to the stress generated by intra/intermolecular interaction and the radial diffusion of TMB from the F127/PDA composite to bulk solution (Figure S6d,e, Supporting Information). After reacting for 1 h, the thickness of mesostructured PDA/F127 composite almost remains constant, and the polymer framework can be further fixed and become more rigid by further polymerization.^[^ ^17^ ^]^ The clear boundary between the PDA and F127 phases around Fe_3_O_4_@*n*SiO_2_ microspheres (Figure S6f, Supporting Information) provides ambiguous evidence for the amphiphilic F127‐directed assembly and polymerization process. By changing the concentration of dopamine in the synthesis solution from 3 to 12 mg L^−1^, a series of Fe_3_O_4_@*n*SiO_2_@PDA/F127 composite microspheres and corresponding MMP‐V with tunable shell thickness of 82–125 nm can be obtained (Figure S7, Supporting Information). It is worth noting that, the presence of TMB is indispensable for this abc‐DIAP synthesis. Without TMB, no micellization of F127 occurs in the synthesis solution. Because considerable amount of ethanol (about 50 v/v%) is present and both PPO and PEO segments of F127 molecules are soluble, F127 molecules can interact with PDA around the Fe_3_O_4_@*n*SiO_2_ without forming ordered mesostructure, and thus only composite particles of Fe_3_O_4_@*n*SiO_2_@PDA/F127 with ill‐defined structure and morphology are obtained (Figure S8, Supporting Information). When hydrophobic TMB is added, stable F127/TMB/ethanol–water nanoemulsion can be readily formed (Figure S9, Supporting Information), and therefore, interface coassembly and polymerization can occur via hydrogen‐bond interaction among silanol groups in Fe_3_O_4_@*n*SiO_2_ surface, PDA oligomers and PEO segments of F127, forming a layer of TMB‐swelled F127/PDA composite on the magnetic seeds (Scheme 1). Furthermore, the mesopore size can be well controlled by adjusting the dosage of TMB.^[^ ^43^ ^]^ Notably, other amphiphilic block copolymers even those containing highly immiscible blocks (e.g., lab‐made PEO_108_‐*b*‐PS_210_) can be employed in this abc‐DIAP process. PEO‐*b*‐PS block copolymers can spontaneously form compact spherical micelles with TMB assistance, and they can coassemble with PDA around Fe_3_O_4_@*n*SiO_2_ seeds, forming a uniform shell made of closely packed PEO‐*b*‐PS/PDA spherical composite micelles. The uniform spherical mesopores are mainly due to the ultralong hydrophobic PS chains which tend to form stable spherical micelles (Figure S10, Supporting Information).

Based on the above results, we proposed a nanoemulsion assisted interface assembly mechanism for the synthesis of core–shell magnetic mesoporous polymer/carbon microspheres. First, TMB is well dispersed in water and form a uniform and stable spherical nanoemulsion due to the stabilization effect of amphiphilic block copolymers (F127/PEO‐*b*‐PS) at the interface between TMB droplets and H_2_O, generating spherical F127/TMB micelles with TMB as the liquid “pool”, PPO segments dissolved in TMB phase and PEO reaching out to the bulk ethanol–water solution. After adding Fe_3_O_4_@*n*SiO_2_ seeds and dopamine, both Fe_3_O_4_@*n*SiO_2_ colloids and spherical F127/TMB micelles can adsorb dopamine molecules via hydrogen bond interaction. The spherical DA/F127/TMB composite micelles tend to deposit onto the magnetic colloids to decrease the interface energy of the system. Simultaneously, dopamine undergoes oxidative polymerization under the catalysis of ammonium hydroxide and co‐assembles with the micelles by virtue of hydrogen bond interaction between EO segments and phenolic hydroxyl groups of dopamine. The interaction between DA/F127/TMB composite micelles can create curved interfaces at neighboring micelles around magnetic colloids, and the concentration of DA molecules at the interfaces increases, which accelerates the local polymerization of DA. It can induce the composite micelles to transform into 1‐D cylindrical micelles that further assemble into ordered mesostructure with cylindrical micelles aligned perpendicularly to the magnetic colloids’ surface (**Scheme** **2**). After the mesostructures are fixed via further intra/intermolecular interaction of PDA^[^ ^52^, ^53^ ^]^ and F127 surfactants are removed, perpendicularly aligned mesopore channels are obtained. In the synthesis, TMB plays two key roles. First, it helps to form uniform F127‐stabilized water/oil nanoemulsion, which favors the assembly of mesostructures at the interface of magnetic colloids by controlling the molecule–colloid–nanoemulsion interaction. Second, it can swell the micelles by hydrophobic–hydrophobic interaction, thus enlarging the final mesopore size. As TMB content increases, the composite micelles’ size can be dramatically enlarged, thus generating larger mesopores in the PDA shell.^[^ ^1^ ^]^ In the absence of TMB, all F127 molecules are well dissolved in the water–ethanol solution without microphase separation, and therefore, composite micelles are not formed and the nonporous PDA shell is generated.

**Scheme 2:**
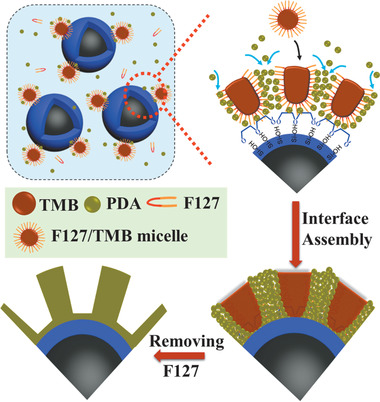
Illustration of the formation mechanism for MMP‐V microspheres through amphiphilic block copolymer directed interface co‐assembly and polymerization (abc‐DIAP).

It is well known that PDA is a recently emerged hydrophilic functional polymer and has been widely exploited for various applications such as drug delivery,^[^ ^54^ ^]^ photothermotherapy,^[^ ^55^, ^56^ ^]^ tissue engineering,^[^ ^53^, ^57^ ^]^ and environmental protection.^[^ ^58^ ^]^ The obtained magnetic mesoporous PDA (MMP‐V) microspheres in this study possess integrated merits of excellent dispersibility, biocompatible surface, abundant functional groups (e.g., catechol —OH groups, amino groups) and magnetic separability, and are particularly suitable for biological application. Separation and enrichment of low‐abundance small‐sized (or low‐molecular weight) proteins from complex biosamples are of great significance to analyze and understand pathological and biomedical systems because low‐abundance small‐sized proteins have potential applications in biomarkers and signal molecules.^[^ ^59^ ^]^ In this study, the obtained MMP‐V microspheres (**Figure** **3**a) with mesopores of 11.9 nm were employed as the absorbent to investigate their adsorption performance by using bovine serum albumin (*M*_W_ = 66 400 Da, size = 5.0 × 7.0 × 7.0 nm^3^) and cytochrome c (Cyt.C, size = 2.6 × 3.2 × 3.3 nm^3^) as model proteins.

**Figure 3:**
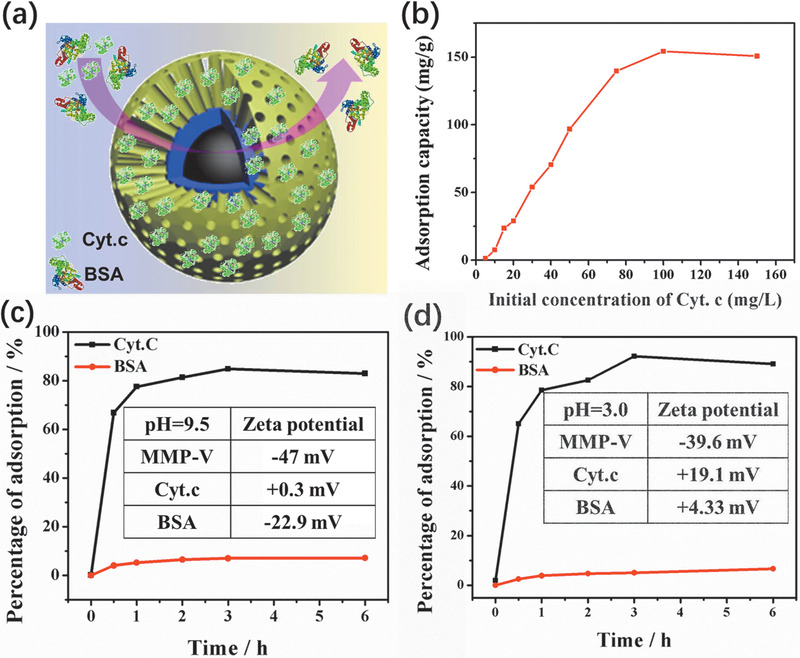
a) Schematic illustration of size‐selective protein adsorption by MMP‐V microspheres; b) the saturated adsorption curve of Cyt.C by MMP‐V microspheres; the size‐selective protein adsorption performance in Cyt.C/BSA mixtures by MMP‐V microspheres in c) alkaline and d) acidic buffers; the inset is the corresponding texture of zeta potential.

To study their adsorption capacity, an equal amount of absorbents is used to incubate in Cyt.C solutions of different concentrations. The solution was adjusted to pH 9.5 where the magnetic MMP‐V absorbents and Cyt.C are negatively and slightly positively charged with zeta potential of −47 and 0.3 mV, respectively. Such a condition is favorable for host–guest interaction during the adsorption process. With the increase of Cyt.C concentration, the adsorption amount of Cyt.C by MMP‐V microspheres continuously increases and reaches a maximum value of 152.5 mg g^−1^ at the Cyt.C concentration of 100 mg L^−1^ (Figure 3b). Such a large adsorption capacity is mainly due to the large and accessible mesopores, high specific surface area and pore volume. To further study their adsorption behavior in mixed protein solutions, equivalent absorbents are incubated in a mixed solution of Cyt.C (100 mg L^−1^) and BSA (100 mg L^−1^) at pH 9.5. As shown in Figure 3c, after 2 h incubation and magnetic separation, about 85% Cyt.C can be adsorbed and separated from the solution by the absorbent, and by contrast, little adsorption for BSA (<10%) is observed. Such an adsorption difference might be due to the size‐selective adsorption towards Cyt.C (about 3.0 nm) driven by electrostatic attraction, and the electrostatic repulsion between negatively charged BSA and the absorbents (Figure 3c inset). To eliminate the influence of electrostatic interaction, a similar adsorption experiment is performed in the mixed solution of Cyt.C (100 mg L^−1^) and BSA (100 mg L^−1^) at pH 3.0, in which BSA shows a positively charged surface while MMP‐V microspheres remain negatively charged. MMP‐V microspheres still show poor BSA adsorption performance with a negligible adsorption capacity of 7.1 mg g^−1^. It indicates that the adsorption performance is mainly due to the size‐selectivity by the uniform mesopores rather than the surface electrostatic interaction (Figure 3d). Therefore, these MMP‐V microspheres with good dispersibility and uniform radial pore channels are ideal candidates absorbents for applications in bioenrichment or sample pretreatment.

Polydopamine has been also used as an important carbon source because it can be carbonized into N‐containing carbon materials that show unique properties for various applications.^[^ ^40^, ^41^, ^42^, ^43^ ^]^ Herein, through direct pyrolysis treatment of the as‐made Fe_3_O_4_@*n*SiO_2_@PDA/F127 composites in nitrogen, core–shell magnetic mesoporous carbon microspheres (MMC‐V) with a mean particle size of ≈380 nm and uniform mesopores are obtained (**Figure** **4**a,b). The carbon shell (≈90 nm) is slightly thinner than the PDA shell (≈100 nm) in the MMP‐V due to the structure shrinkage during carbonization of PDA. Nitrogen sorption isotherm confirms the buckled large cylindrical mesoporous structure and the pore size distribution reveals a mean diameter of ≈12.7 nm (Figure 4c,d). The BET surface area and total pore volume are calculated to be 251.7 m^2^ g^−1^ and 0.17 cm^3^ g^−1^ (Table S1, Supporting Information), respectively. The wide‐angle X‐ray diffraction (XRD) patterns (Figure 2c) show the typical diffraction peaks of Fe_3_O_4_ and amorphous carbon, indicating the well‐retained magnetite phase in the whole synthesis process. XPS spectra (Figure S4d–f, Supporting Information) shows enhanced C and N peaks than that of MMP‐V due to the further carbonization of PDA. An enhanced peak density of C—N bond and distinct graphitic N can be seen in high‐resolution C 1s profile and N 1s profile, respectively, indicating a high C—N species density and a high degree of graphitization after calcination.

**Figure 4:**
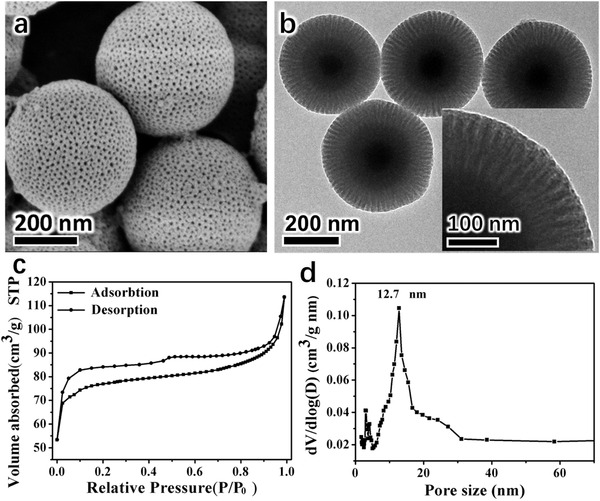
a) SEM, b) TEM images, c) nitrogen adsorption–desorption isotherm and d) pore size distribution of MMC‐V microspheres.

Noble metal nanoparticles (e.g., Au, Pd, Pt NPs) possess remarkable catalytic activity toward various organic reactions, and they have been widely used as industrial catalysts. However, they are prone to lose activity due to the aggregation in the liquid reaction system, and after the reaction, it is difficult to separate and recycle these ultrafine noble NPs from solutions. In this study, encouraged by the convenient magnetic separation, good stability, large surface area and open mesoporous structure of mesoporous carbon shell, the MMC‐V microspheres are employed to immobilize Au nanoparticles in mesopore channels for heterogeneous catalysis. Compared to traditional magnetic mesoporous silica carriers, the MMC have an excellent chemical stability and hydrothermal stability, therefore demonstrate a desirable heterogeneous catalyst supports since silica easily collapses in catalysis system involved with high‐temperature aqueous or alkaline solution. TEM images and size distribution histogram (Figures S11a,b and S12, Supporting Information) indicate ≈3.7 nm sized Au NPs are uniformly distributed in the mesopore channels of the obtained Au@MMC‐V microspheres (the Au content is about 0.35 wt% according to ICP analysis) through an in situ reduction method.^[^ ^36^ ^]^ High‐resolution TEM image reveals clear crystal lattices of Au NPs. Selected area electron diffraction (SAED) patterns (Figure S11c, Supporting Information) exhibit the spotty diffraction rings ascribed to the excellent crystallinity and dispersion of Au particles. No aggregates of Au particles are observed owing to the mesopore channel confinement. The energy‐dispersive X‐ray (EDX) mapping profile (Figure S11d, Supporting Information) of Au element further confirms the uniform distribution of Au NPs in the mesoporous carbon shell. XPS spectra (Figure S13, Supporting Information) confirm the presence of Au and the chemical bonds and valence of C and N species in high‐resolution C 1s and N 1s profiles well remain after Au immobilization. High‐resolution Au 4f profile confirms two typical peaks of Au^0^ 4f_5/2_ and Au^0^ 4f_7/2_.

To study their catalysis performance, styrene epoxidation by the obtained Au@MMC‐V microspheres was carried out under argon atmosphere using *tert*‐butylhydroperoxide (TBHP) as an oxidant at 82 °C (**Figure** **5**a). Gas chromatography‐mass spectroscopy (GC‐MS) measurements indicate that, apart from the main product styrene oxide, traces of by‐products of benzaldehyde and benzoic acid were also detected. As shown in Figure 5b, the conversion of styrene can achieve around 90% within 25 h. The selectivity toward styrene oxide remains above 80% and slightly decreases with the prolonged reaction time due to overoxidation. The catalytic activity and selectivity are much better than the previously reported results,^[^ ^35^, ^36^ ^]^ mainly due to the high surface area for uniformly and stably dispersing Au NPs, large and open mesoporous structure for the diffusion of reactants and product. Moreover, the hydrophobic and stable carbon framework can enhance the interaction of styrene and catalysts. Thanks to the convenient separation and purification of catalysts by a magnet (≈2000 Gauss), Au@MMC‐V can be easily recycled for reuse. As shown in Figure 5c, the conversion of styrene and selectivity of styrene oxide remain above 80% after reused for six times. TEM characterization (Figure S14, Supporting Information) indicates that the recycled Au@MMC‐V catalysts have excellent stability with well retained and highly dispersed Au NPs in the mesoporous carbon framework after using for six times, indicative of good stability of these magnetic separable heterogeneous nanocatalysts.

**Figure 5:**
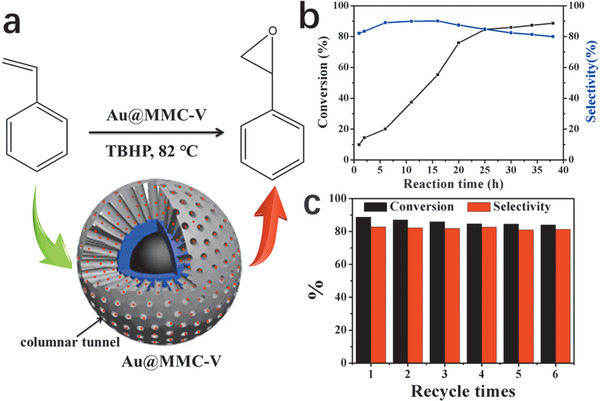
a) Schematic illustration of epoxidation of styrene over Au@MMC‐V microsphere heterogeneous catalysts, b) the conversion of styrene and selectivity toward styrene oxides as a function of reaction time and c) the recycling stability of Au@MMC‐V microspheres in catalyzing the epoxidation of styrene.

In summary, a versatile amphiphilic block copolymer directed interface co‐assembly and polymerization (abc‐DIAP) method is developed for controllable synthesis of core‐shell magnetic mesoporous polydopamine microspheres and their carbon‐based derivatives. The rational manipulation of hydrogen‐bond interactions between block copolymers, dopamine, and Fe_3_O_4_@*n*SiO_2_ colloids facilitates the multi‐component co‐assembly and interfacial polymerization of dopamine and formation of regular core‐shell structures with highly integrated functionalities of magnetic materials, biocompatibility and hydrophilicity of PDA, highly accessible uniform mesopores and high specific surface area. As a result, the obtained magnetic mesoporous PDA microspheres exhibit high adsorption capacity (152.5 mg g^−1^) for Cyt.C and superior selective adsorption of Cyt.C in mixed protein solutions by virtue of the unique size‐exclusion effect of uniform mesopore channels. Through direct pyrolysis treatment and in situ immobilization of Au NPs, the obtained magnetic mesoporous carbon microspheres with stably immobilized Au nanoparticles were demonstrated to be excellent heterogeneous catalysts for epoxidation of styrene with high selectivity (90.1%) and conversion (88.6%). Considering the versatility of this innovative synthesis, it is expected that such an abc‐DIAP method can be extended to construct various functional mesoporous polymers, carbon derivatives, or polymer–inorganic composites, carbon‐based composites for various applications including biomedicine, energy storage, and smart separation systems.

## Conflict of Interest

The authors declare no conflict of interest.

## Supporting information

Supporting Information
