# Supplementary material for: Interface Coassembly and Polymerization on Magnetic Colloids: Toward Core–Shell Functional Mesoporous Polymer Microspheres and Their Carbon Derivatives
Source: Adv Sci (Weinh). 2020 Apr 30;7(12):2000443. doi: 10.1002/advs.202000443 (PMC7312473; doi:10.1002/advs.202000443)
Supplement: Supplementary file 1 — Supporting Information [file ADVS-7-2000443-s001.pdf]

Supplementary Information

Interface Co-assembly and Polymerization on Magnetic Colloids: Toward Core-shell Functional Mesoporous Polymer Microspheres and Their Carbon Derivatives

Panpan Pan [a], Tong Zhang [a], Qin Yue [b,*], Ahmed A. Elzatahry [c], Abdulaziz Alghamdi [d], Xiaowei Cheng [a], Yonghui Deng [a,e,*]

[a] Department of Chemistry, Department of Gastroenterology, Zhongshan Hospital of Fudan University, State Key Laboratory of Molecular Engineering of Polymers, Fudan University, Shanghai 200433, China

[b] Institute of Fundamental and Frontier Sciences, University of Electronic Science and Technology of China, Chengdu, 610051, China

[c] Materials Science and Technology Program, College of Arts and Sciences, Qatar University, PO Box 2713, Doha, Qatar

[d] Department of Chemistry, College of Science, King Saud University, Riyadh 11451, Saudi Arabia

[e] State Key Lab of Transducer Technology, Shanghai Institute of Microsystem and Information Technology, Chinese Academy of Sciences, Shanghai 200050, China

*E-mail: Yonghui Deng, yhdeng@fudan.edu.cn; Qin Yue, qinyue@uestc.edu.cn

Keywords: Core-shell, Magnetic materials, Mesoporous polydopamine, Mesoporous carbon, Interface coassembly.

Experimental section

Chemicals

$FeCl_3 \cdot 6H_2O$, trisodium citrate, sodium acetate, ethylene glycol, ethanol, NaOH, $NaHCO_3$, NaH_2PO_4, HCl concentrated ammonia solution (28 wt%), tetraethyl orthosilicate (TEOS), dopamine hydrochloride, Triblock poly(ethylene oxide)-b-poly(propylene oxide)-b-poly(ethylene oxide), (Pluronic F127, $PEO_{106}PPO_{70}PEO_{106}$, Mav = 12600), 1,3,5-trimethyl benzene (TMB), and Tetrahydrofuran (THF) (> 99%), Bovine serum albumin (BSA, MW. 66,000 Da, 5.0×7.0×7.0 nm^3), cytochrome c (Cyt.C, MW. 13,750 Da, 2.6×3.2×3.3 nm^3) were purchased from Sigma-Aldrich. Sodium borohydride ($NaBH_4$), chloroauric acid tetrahydrate ($HAuCl_4 \cdot 4H_2O$) were purchased from the Aladdin Company. Monomethyl poly (ethylene oxide) (Mw: 5000 $g \cdot mol^{-1}$) was purchased from Aldrich. Copper (I) bromide (CuBr), styrene, pyridine, and Al_2O_3 were purchased from Shanghai Chemical Reagent Co. Ltd. N, N, N′, N″, N″-Pentamethyl diethylenetriamine was purchased from Acros. Acetylacetone, THF, anhydrous ethyl ether, and petroleum ether (30-60 °C, AR) were purchased from Sino-Pharm Chemical Reagent Co. Ltd. 4-nitrophenol (4-NP, 99.0 wt%) and anhydrous ethanol (99.7 wt%) were purchased from Shanghai Chemical Company. Deionized water was used for all experiments. All chemicals were used as received without any further purification.

Synthesis of Fe_3O_4@*n*SiO_2 Microspheres

Fe_3O_4 particles with a mean diameter of ~100 nm were synthesized as we reported previously.[1] An aqueous dispersion of Fe_3O_4 particles (2 mL, 40 g/mL) was added to a three-neck round bottom flask with ethanol (105 mL), H_2O (35 mL) and concentrated ammonia solution (2.0 mL) under the mechanical stirring (200 rpm) at 30 ^{o}C. After stirring for 30 min, 3.0 ml of TEOS (2.79 g) was added dropwise and the reaction was allowed to proceed for 8 h with continuous mechanical stirring. After that, a magnet was used to separate and collect the core-shell Fe_3O_4@*n*SiO_2 microspheres, followed by washing three times with ethanol and water, respectively.

Synthesis of Core-Shell Magnetic Mesoporous Polydopamine Microspheres with vertically aligned cylindrical mesopores (MMP-V)

In a typical synthesis, 100 mg of F127 and 0.1 mL TMB were gradually dissolved in ethanol (4.7 mL) and deionized water (5 mL) by ultrasonication. The resultant transparent solution gradually turned into pale blue due to the micellization of F127 block copolymers [2]. Then, 0.3 ml of ethanol solution containing 20 mg of Fe_3O_4@*n*SiO_2 microspheres and 120 mg of DA were added with stirring (280 rpm) at 25 ºC. After continuous stirring for 1h, 0.06 mL $NH_3 \cdot H_2O$ (28 wt%) was dropwise added, and the reaction was allowed to proceed for 2 h under continuous mechanical stirring. The as-made samples (Fe_3O_4@*n*SiO_2@PDA/F127 microspheres) were collected by magnetic separation, followed by washing with deionized water and ethanol three times.

After collected, the as-made samples were redispersed in 80 mL acetone of and refluxed at 80 °C overnight to remove F127 templates. The extraction was repeated 6 times and then thoroughly washed with ethanol. After vacuum drying, core-shell magnetic mesoporous polydopamine microspheres (Fe_3O_4@*n*SiO_2@mPDA) with vertically aligned cylindrical mesopores were obtained.

For the synthesis of core-shell magnetic mesoporous polydopamine nanochains with vertically aligned cylindrical mesopores, an applied magnetic field was introduced into the interface coating of silica as we reported previously. [3] Then, a layer of PDA/F127 was coassembled and deposited onto the Fe_3O_4@*n*SiO_2 nanochain with the same method as described above. After acetone extraction to remove F127, Fe_3O_4@*n*SiO_2@mPDA nanochains were obtained. To adjusting the mPDA shell thickness, the amount of dopamine in the synthesis was changed from 30 to 120 mg.

Synthesis of Core-Shell Magnetic Mesoporous Carbon Microspheres with vertically aligned cylindrical mesopores (MMC-V)

After drying at 60 ^oC in vacuum, the as-made sample (Fe_3O_4@*n*SiO_2@PDA/F127 microspheres) was calcined at 300 ^oC in N_2 atmospheres at a heating rate of 1 $^oC\cdot min^{-1}$ for 1 h, and further raised to 550 ^oC at a heating rate of 1 $^oC\cdot min^{-1}$ and kept for 2 h, generating core-shell magnetic mesoporous carbon spheres (Fe_3O_4@*n*SiO_2@mCarbon) with vertically aligned cylindrical mesopores (denoted as MMC-V).

Synthesis of Core-Shell Magnetic Mesoporous Polydopamine Microspheres with spherical mesopores (MMP-S)

For the synthesis of Fe_3O_4@*n*SiO_2@PEO-*b*-PS/PDA, the amphiphilic PEO-*b*-PS diblock copolymers with a composition of PEO_{108}-*b*-PS_{210} (Mn = 26544 g·mol^{-1}, PDI = 1.09) were prepared according to previously reported procedures. [4] The synthesis procedure is similar to that for MMP-V spheres, except that the reaction solution was ethanol/H_2O/THF (The volume ratio was 1:2:1) instead of ethanol/H_2O (The volume ratio was 1:1), and meanwhile the amount of template was 20 mg of PEO_{108}-*b*-PS_{210} instead.

Protein adsorption.

Firstly, standard curves were fitted according to the characteristic UV-vis absorption peak intensity of various concentrations (5-100 mg/L) of protein buffer solution at a wavelength of 409 nm and 285 nm for Cyt.C and at for BSA, respectively (shown as below).

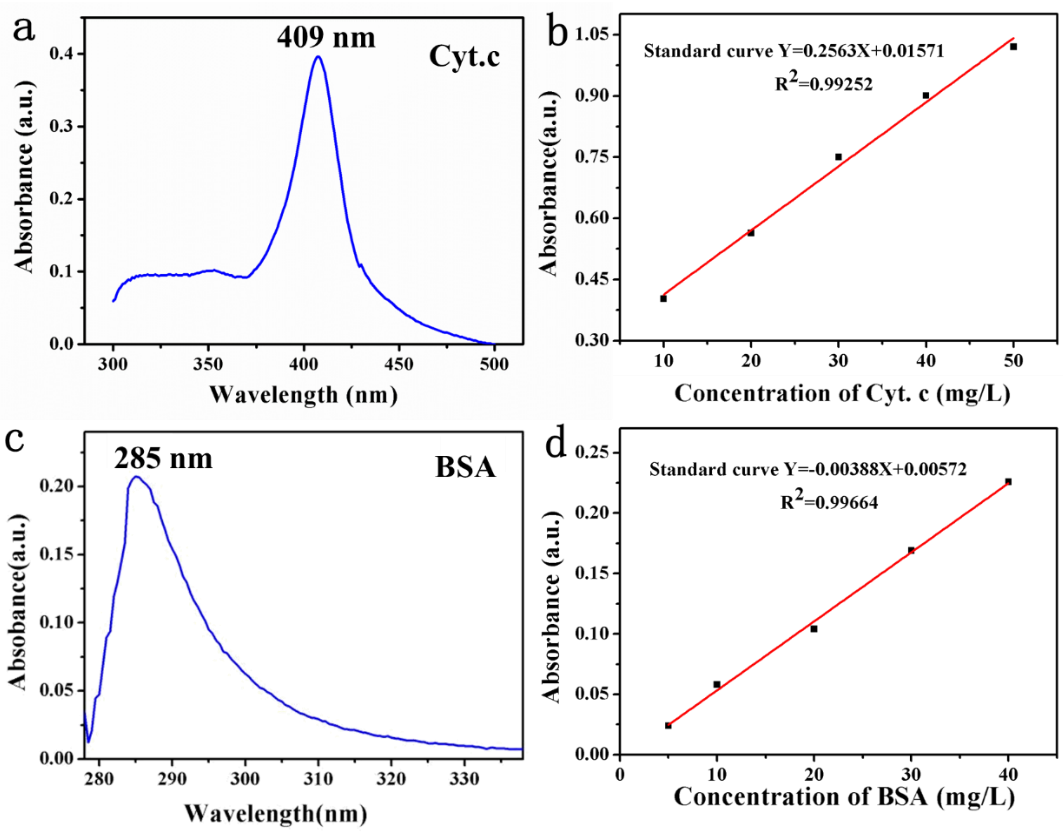

The characteristic UV-vis spectrum (a, c) and the corresponding standard curves (b, d) of Cyt.C (a, b) and BSA (c, d) in a buffer (pH 9.5).

The saturated adsorption capacity of Cyt.c by MMP-V was studied through monitoring the adsorption capacity in various initial concentrations (5-150 mg/L) of Cyt.C solution in $NaOH/NaHCO_3$ buffer (pH 9.5). 0.5 mg of MMP-V was added into the Cyt.C/NaOH/ $NaHCO_3$ buffer (pH 9.5) solution and the mixture was then incubated in a shaker at room temperature for above 6 h. Then MMP-V was separated from the mixture using an external magnet and washed three times with buffer. UV-vis spectroscopy measurement was employed to determine the amount of retained Cyt.C in the magnetic supernatant and washing buffer by monitoring the characteristic peak intensity of 409 nm. The amount of adsorbed proteins was calculated by subtracting the free proteins in the magnetic supernatant and washing buffer after a specific time from the initial amount of proteins.

For size-selective protein adsorption measurement, typically, 0.5 mg of MMP-V was transferred to the protein $NaOH/NaHCO_3$ buffer (pH 9.5) solution containing 5 mL of Cyt.C or 5 mL of BSA with the same concentration (100 mg/L). The mixture was then incubated in a shaker at room temperature for above 6 h. Then MMP-V was separated from the mixture using an external magnet and washed three times with buffer. UV-vis spectroscopy measurement was employed to determine the retained protein of the magnetic supernatant and washing buffer by monitoring the characteristic peak intensity of 409 nm and 285 nm for Cyt.C and at for BSA, respectively. The amount of adsorbed proteins was calculated by subtracting the free proteins in the magnetic supernatant and washing buffer after a specific time from the initial amount of proteins. Besides the alkaline buffer solution, the size-selective protein adsorption was also measured in NaH_2PO_4/HCl buffer solution (pH 3.0) with the same method.

Synthesis of Au-loaded MMC-V microspheres (Au@MMC-V)

Au nanoparticles were loaded into MMC-V microspheres via an *in situ* reduction method. In a typical synthesis, 20 mg of MMC-V microspheres were dispersed in 10 mL ethanol solution by ultrasonication treatment. Then, 0.8 mL of $HAuCl_4$ (0.2 mg/mL) was added, followed by 0.2 mL of ice-cold, freshly prepared 0.1M of $NaBH_4$ solution. The products were centrifuged after reaction for 2 h, washed with deionized water and ethanol three times, respectively. After drying in a vacuum at 60 ºC, the obtained sample was denoted as Au@MMC-V microspheres.

Catalytic epoxidation of styrene

20 mg of catalyst (Au@MMC-V) was added to a mixture of styrene (2.6 mL, 20 mmol), and acetonitrile (10 mL). The dispersion was bubbled with high purity Ar for 60 min at room temperature. After adding t-butyl hydroperoxide (10.0 g, 76 mmol, 70 wt% in water), the reaction vessel was immersed in an oil bath and heated at 80 ºC. During the reaction process, a minor amount of reaction solution (20 mL) was withdrawn at different time intervals for gas chromatography-mass spectrometer (GC-MS) measurements. After reaction for 40 h, the reaction system was cooled down, and the catalyst was recycled using a magnet, washed 3 times with acetonitrile, and vacuum dried at 40 ºC for reuse.

Measurements and characterization

Field-emission scanning electron microscopy (FESEM) images were collected on the Hitachi model S-4800 field emission scanning electron microscope (Japan). The dried samples were directly used for the observation without any treatment. Transmission electron microscopy (TEM) images were taken with a JEOL 2011 microscope (Japan) operating at 200 kV. For the TEM measurements, the samples were dispersed in ethanol and then dried on a holey carbon film Cu grid. Chemical analysis of the samples was performed by energy-

dispersive X-ray spectroscopy (EDS) in the TEM (FEI Talos F200X, USA) using SuperX silicon drift detectors built-in the pole piece of the objective lens for high-resolution and high-precision elemental mapping. Nitrogen adsorption-desorption isotherms were measured at 77 K with a Micromeritcs Tristar 3000 analyzer. Before measurements, the samples were degassed in vacuum at 180 ºC for 10 h. The Brunauer-Emmett-Teller (BET) method was utilized to calculate the specific surface areas in a relative pressure range from 0.005 to 0.25. By using the Barrett-Joyner-Halenda (BJH) model, the pore volumes and pore size distributions were derived from the adsorption branches of isotherms, and the total pore volumes (V_t) were estimated from the adsorbed amount at a relative pressure P/P_0 of 0.995. Powder X-ray diffraction (XRD) patterns were recorded on a Bruker D4 X-ray diffractometer (Germany) with Ni-filtered Cu K_R radiation (40 kV, 40 mA). X-ray photoelectronic spectroscopy (XPS) spectra were carried out at room temperature by using a JPS-9010TR (JEOL) instrument with an Mg Kα X-ray source. The sample was compressed into films with KBr under reduced pressure, and further characterized via using Fourier transform infrared (FT-IR) spectroscopy (Thermo Nicolet 360, Nicolet, USA). The UV-vis spectra were recorded on a UV-vis spectrometer (Jasco V-550) at 25 °C. The Au content loaded in the symples was carried out by inductively coupled plasma-atomic emission spectrometry (ICP-AES, Varian VISTA-MPX).

References:

[1] J. Liu, Z. Sun, Y. Deng, Y. Zou, C. Li, X. Guo, L. Xiong, Y. Gao, F. Li, D. Zhao, *Angew. Chem. Int. Ed.* **2009**, *48*, 5875.

[2] B. Y. Guan, L. Yu, X. W. Lou, *J. Am. Chem. Soc.* **2016**, *138*, 11306.

[3] L. Wan, H. Song, X. Chen, Y. Zhang, Q. Yue, P. Pan, J. Su, A. A. Elzatahry, Y. Deng, *Adv. Mater.* **2018**, *30*, 1707515.

[4] J. Zhang, Y. Deng, D. Gu, S. Wang, L. She, R. Che, Z. S. Wang, B. Tu, S. Xie, D. Zhao, *Adv. Energy Mater*. **2011**, *1*, 241.

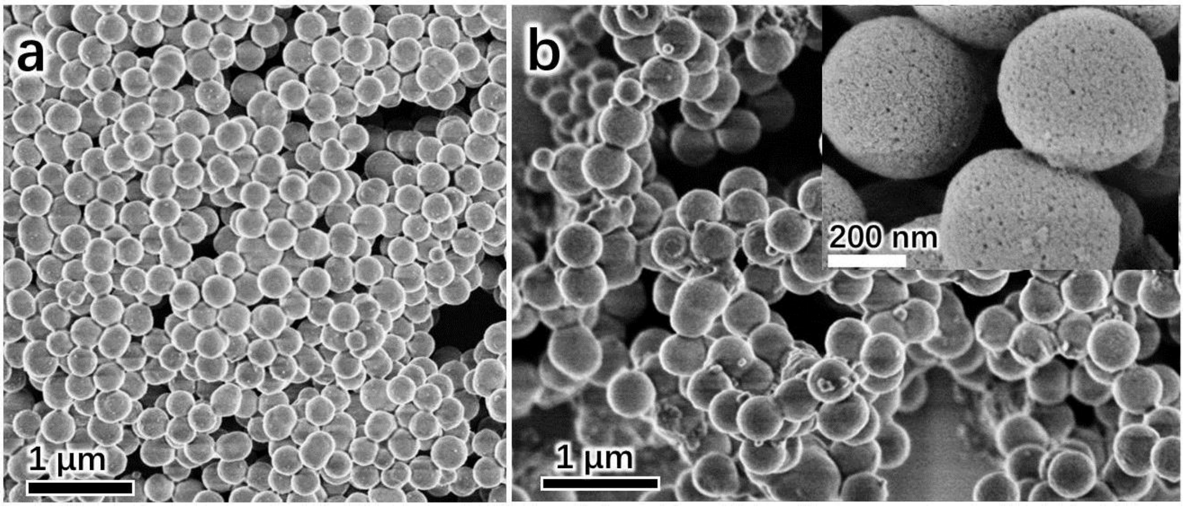

Figure S1. SEM images of (a) Fe_3O_4@*n*SiO_2 and (b) Fe_3O_4@*n*SiO_2@F127/PDA composite microspheres obtained after interface co-assembly of F127 with dopamine and spontaneous oxidative polymerization.

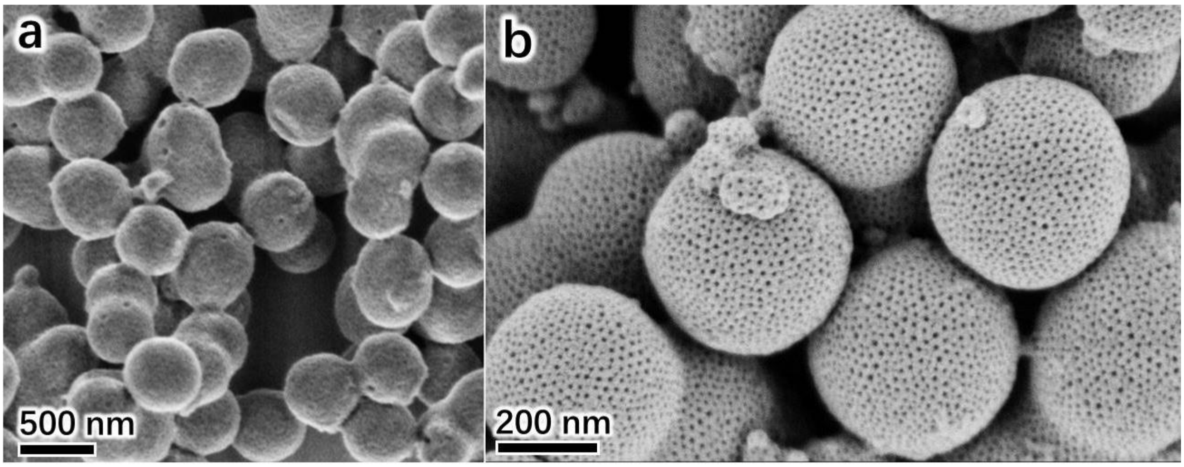

Figure S2. SEM images of MMP-V microspheres obtained by interface coassembly and polymerization on Fe_3O_4@*n*SiO_2 microspheres using F127 as structure-direncting agent for assembly with dopamine.

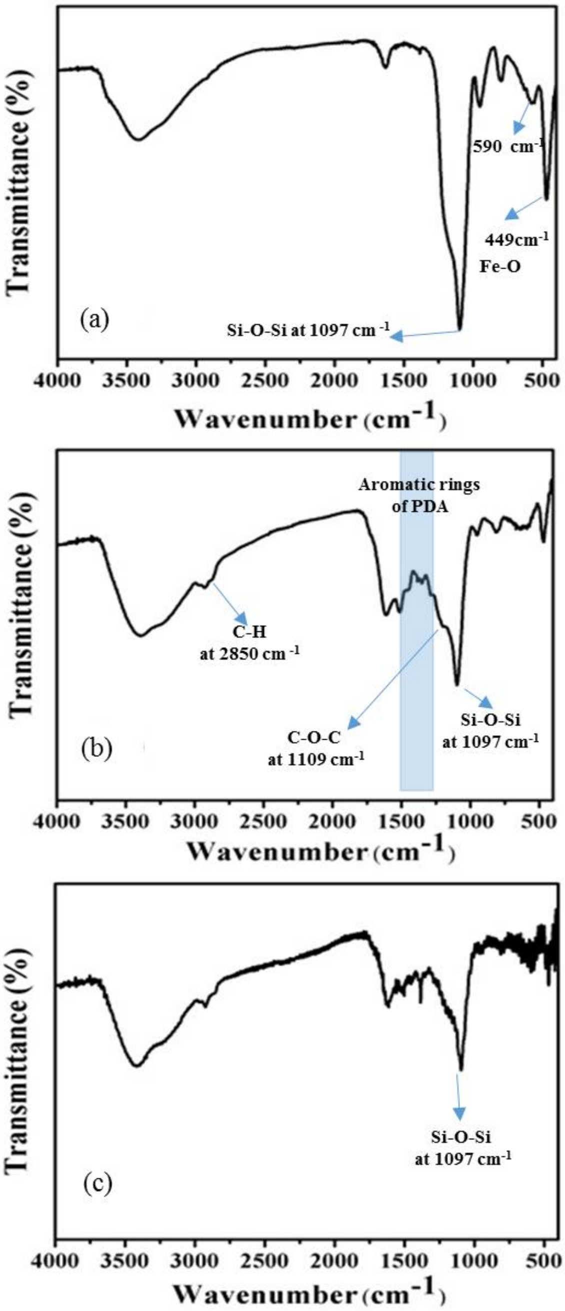

Figure S3. FTIR spectrums of Fe_3O_4@*n*SiO_2 microspheres (a), Fe_3O_4@*n*SiO_2@F127/PDA composite microspheres (b) obtained after interface coassembly of F127 with dopamine and spontaneous oxidative polymerization and MMP-V microspheres (c) obtained after removing F127 templates via acetone extraction from the composite microspheres.

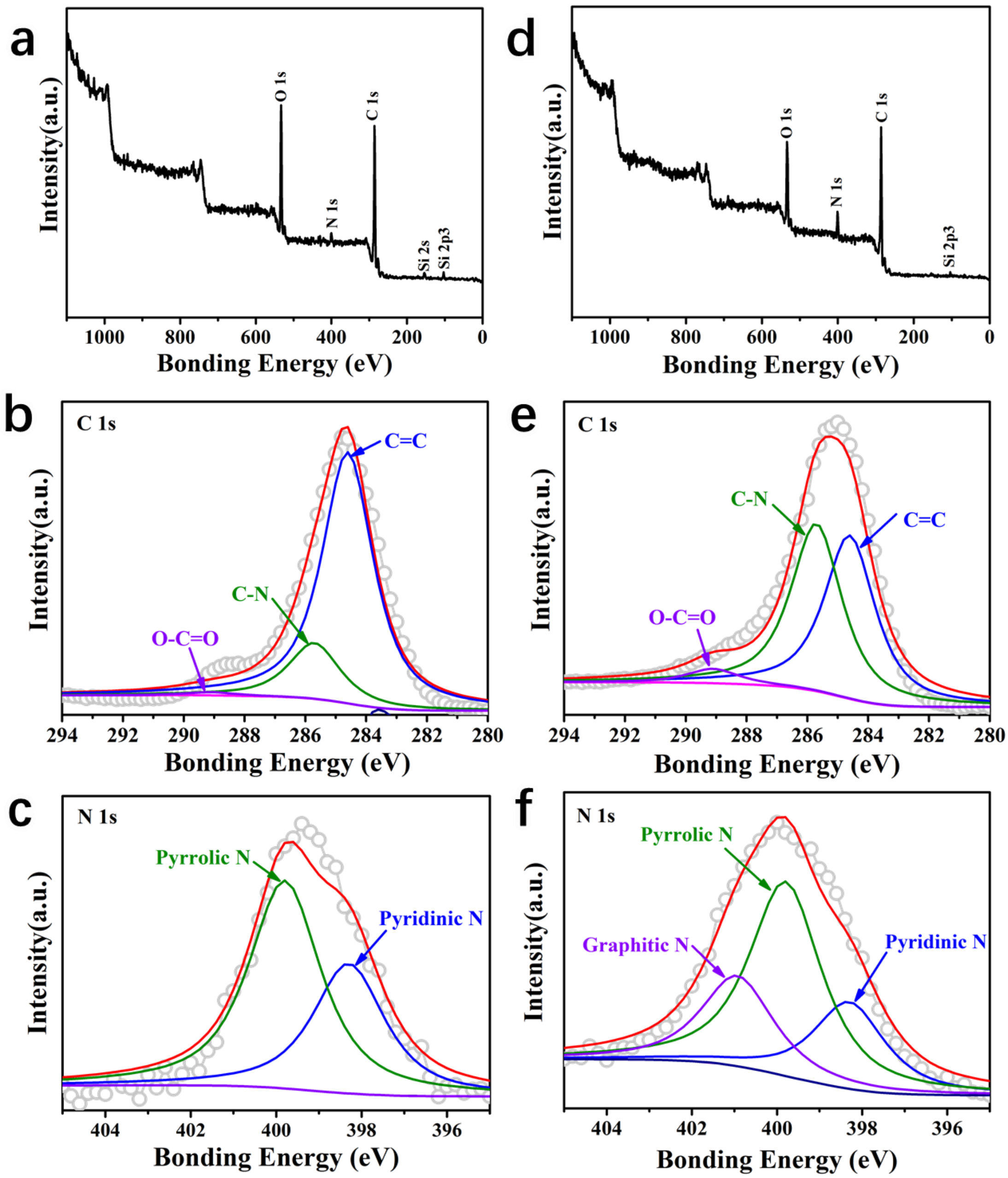

Figure S4. XPS survey spectra (a, d) and high resolution XPS spectra of C 1s (b, e) and N 1s (c, f) for MMP-V (a-c) and MMC-V (d-f) obtained through calcination treatment of MMP-V in nitrogen.

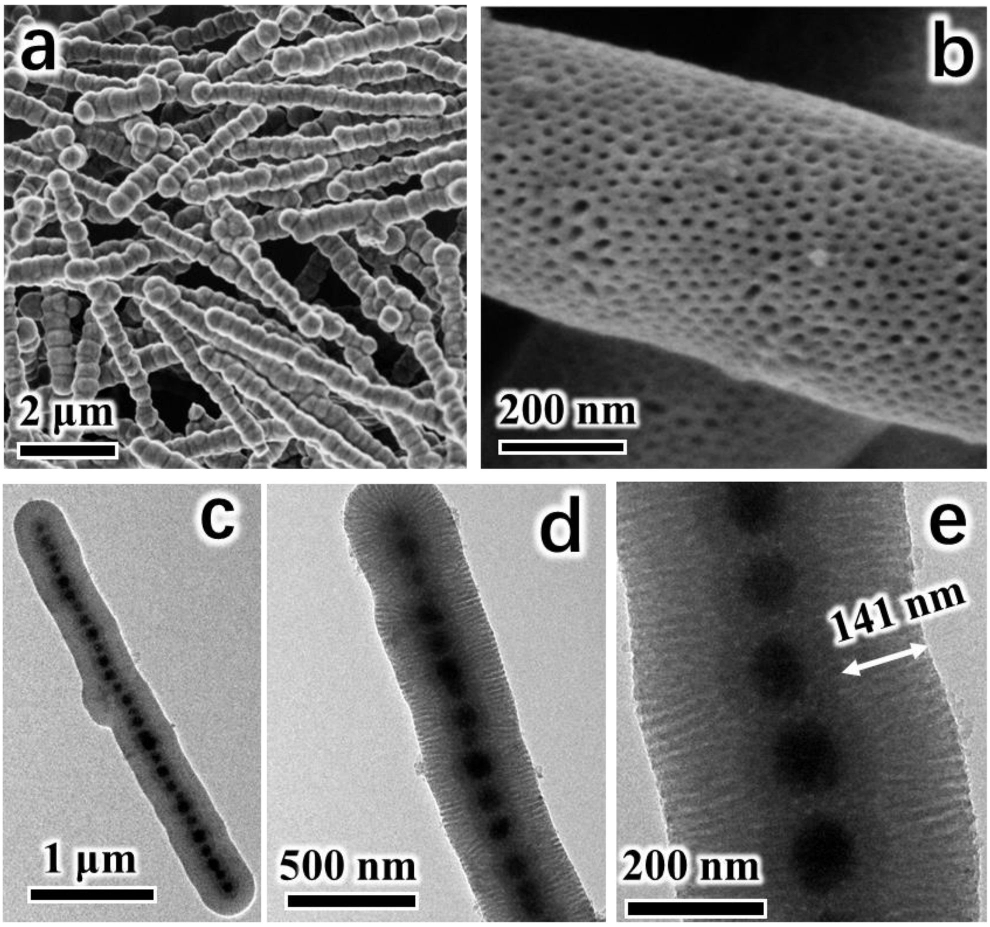

Figure S5 . SEM (a-b) and TEM (c-e) images of Fe_3O_4@*n*SiO_2@mPDA nanochains.

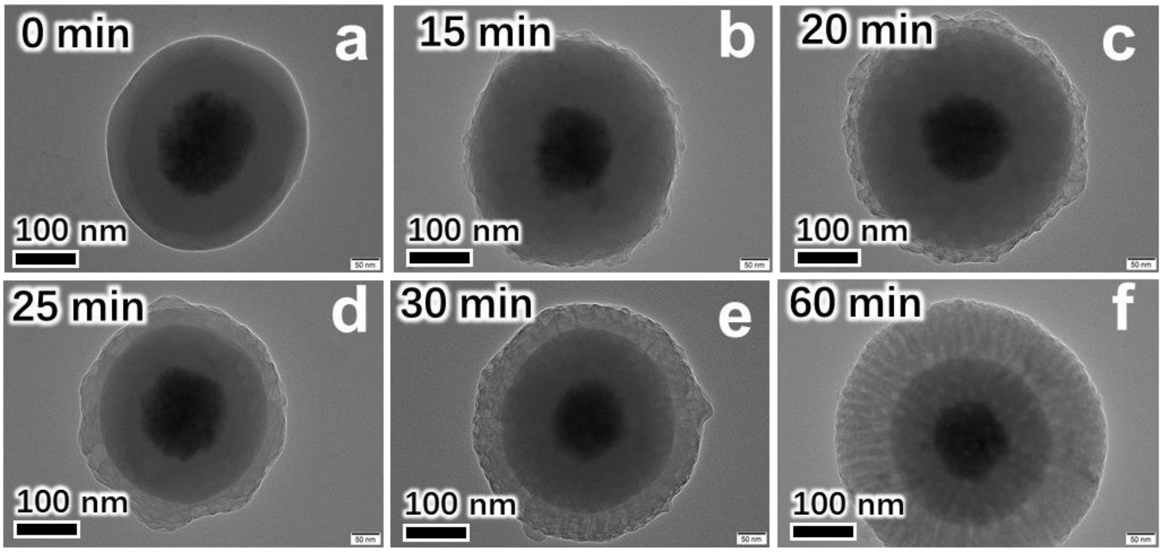

Figure S6. TEM images of intermediate samples of Fe_3O_4@*n*SiO_2@F127/PDA composite microspheres withdrawn during synthesis from the reaction solution

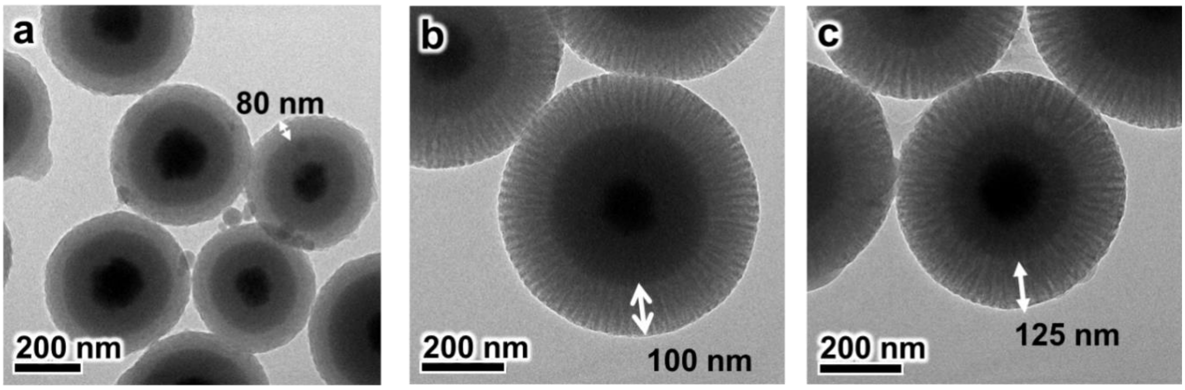

Figure S7. TEM images of MMP-V with various shell thickness (82-125 nm) obtained using different concentration of dopamine.

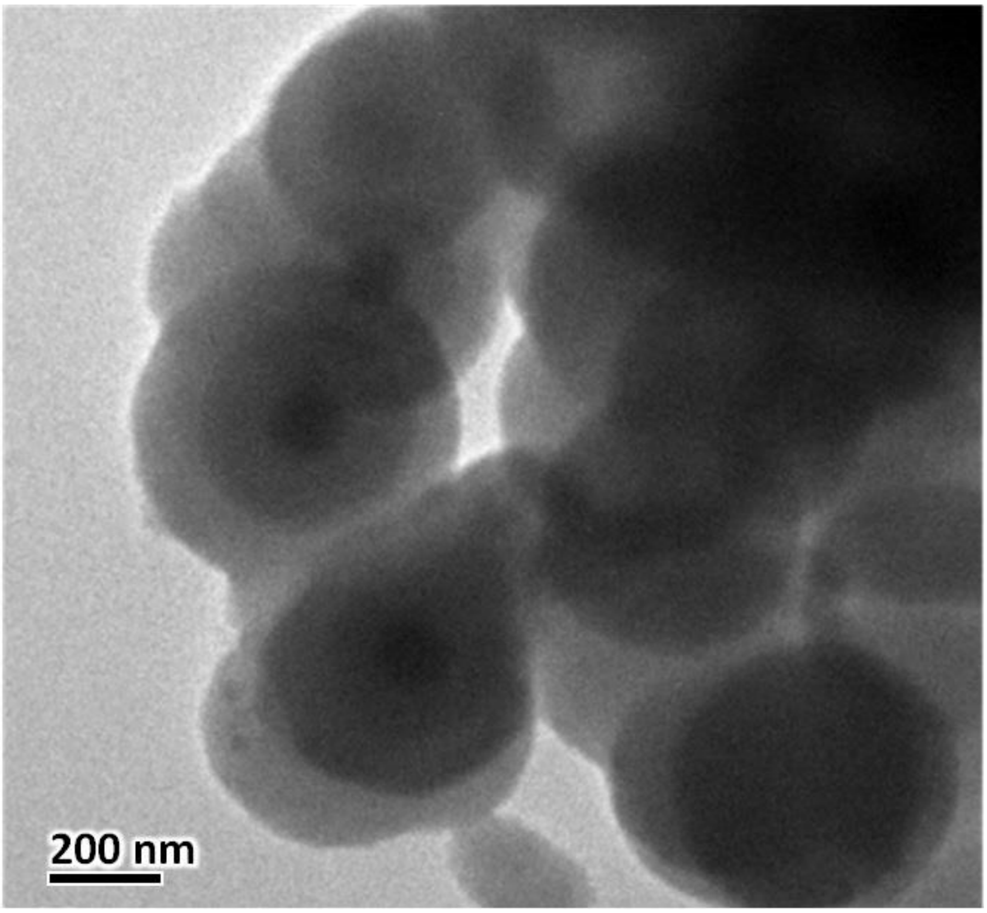

Figure S8. TEM images of core-shell magnetic polydopamine microspheres synthesized in the similar solution for MMP-V microspheres except that F127 molecules were used the interface structure-directing agent in the absence of TMB.

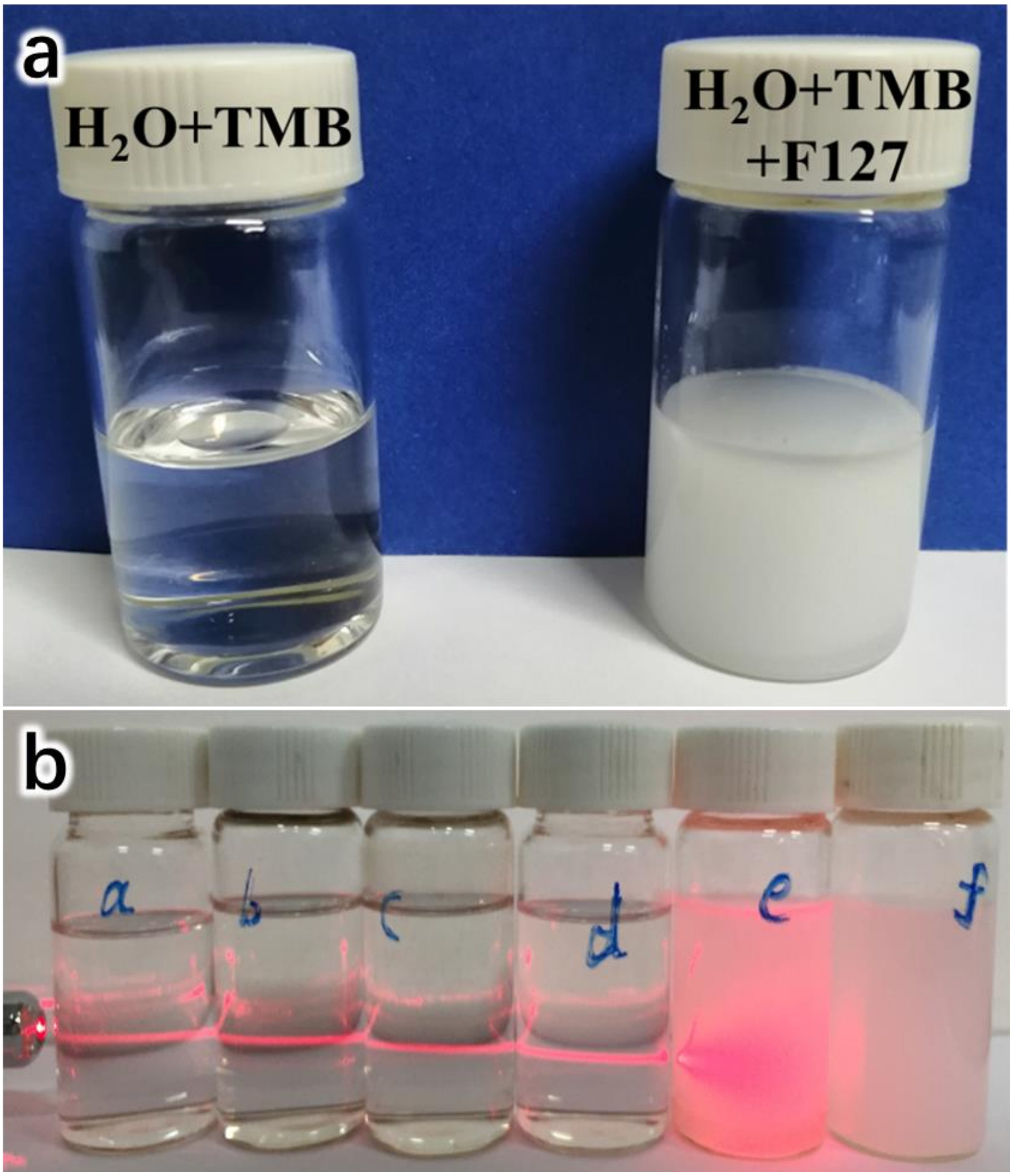

Figure S9. Photographs of H_2O/ethanol/TMB solution in the absence and presence of F127 (a), and H_2O/ethanol/TMB mixture with an increasing amount of TMB from 0.05 to 0.3 mL under 650 nm laser illumination (b).

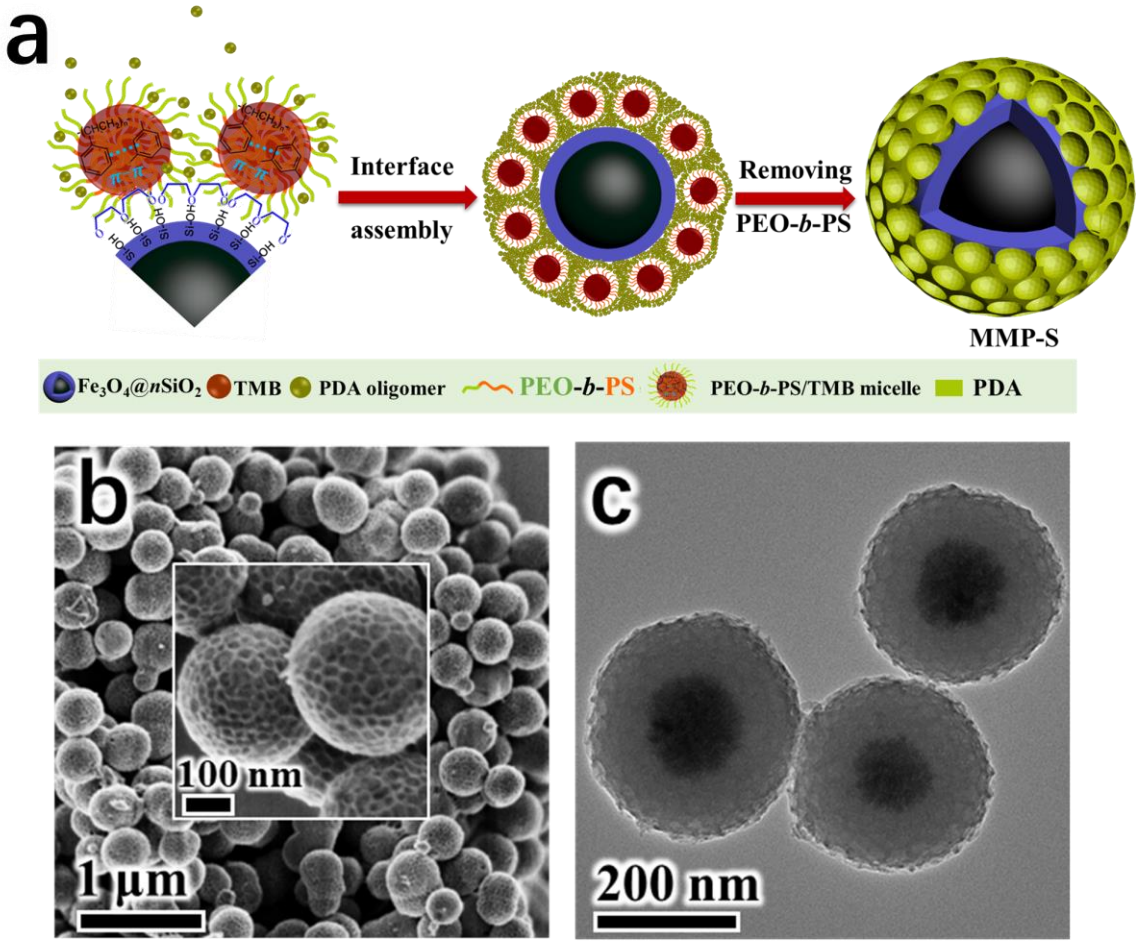

Figure S10. (a) Illustration of the formation mechanism for MMP-S microspheres through amphiphilic block copolymer directed interface co-assembly and polymerization (abc-DIAP); SEM (b) and TEM (c) images of Fe_3O_4@*n*SiO_2@PEO-*b*-PS/polydopamine.

Table S1. Texture properties of the core-shell magnetic mesoporous polymer or carbon spheres templated from F127.

Sample	Surface area [m^2g^{-1}]	Pore volume [cm^3g^{-1}]	Pore size [nm]
MMP-V	235.6	0.16	11.9
MMC-V	251.7	0.17	12.8

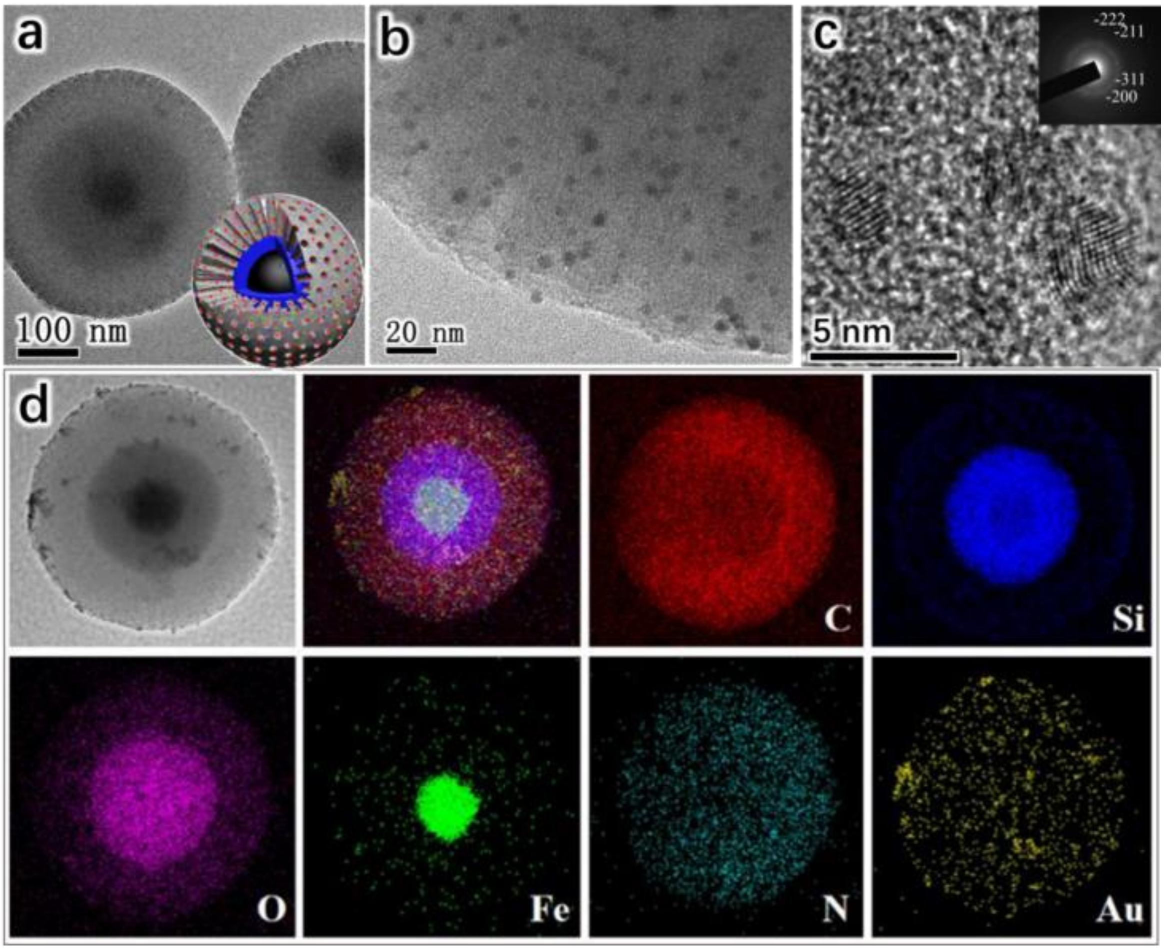

Figure S11. TEM images (a-c) of Au@MMC-V microsphere and energy-dispersive X-ray element mapping (d) of C, Si, O, Fe, N, and Au elements in a single Au@MMC-V microsphere.

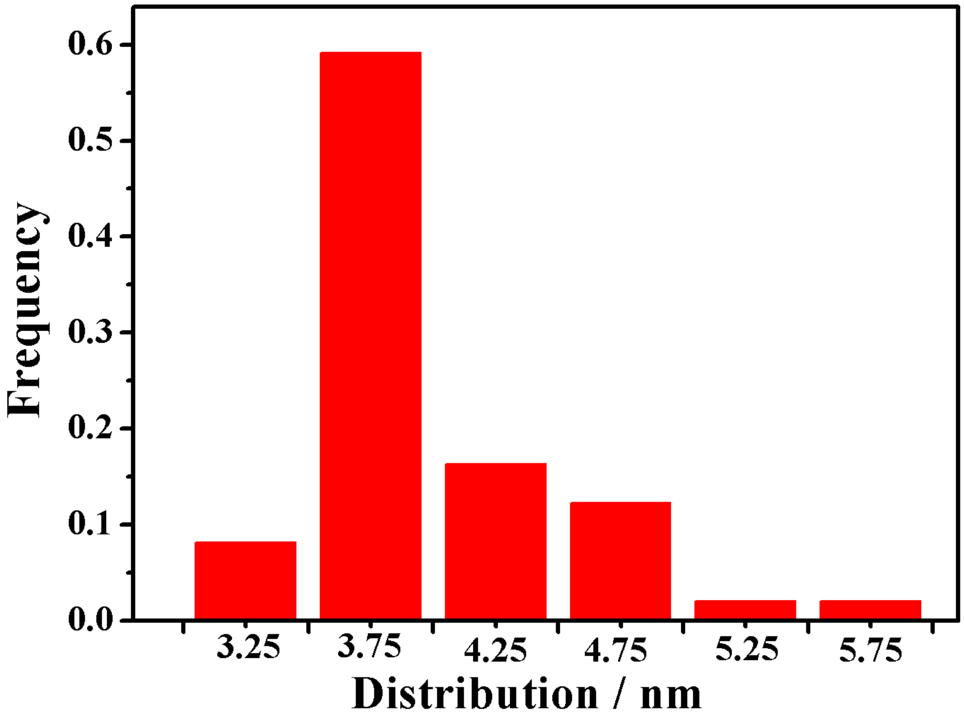

Figure S12. Size distribution histograms for Au nanoparticles immobilized in the MMC-V microspheres.

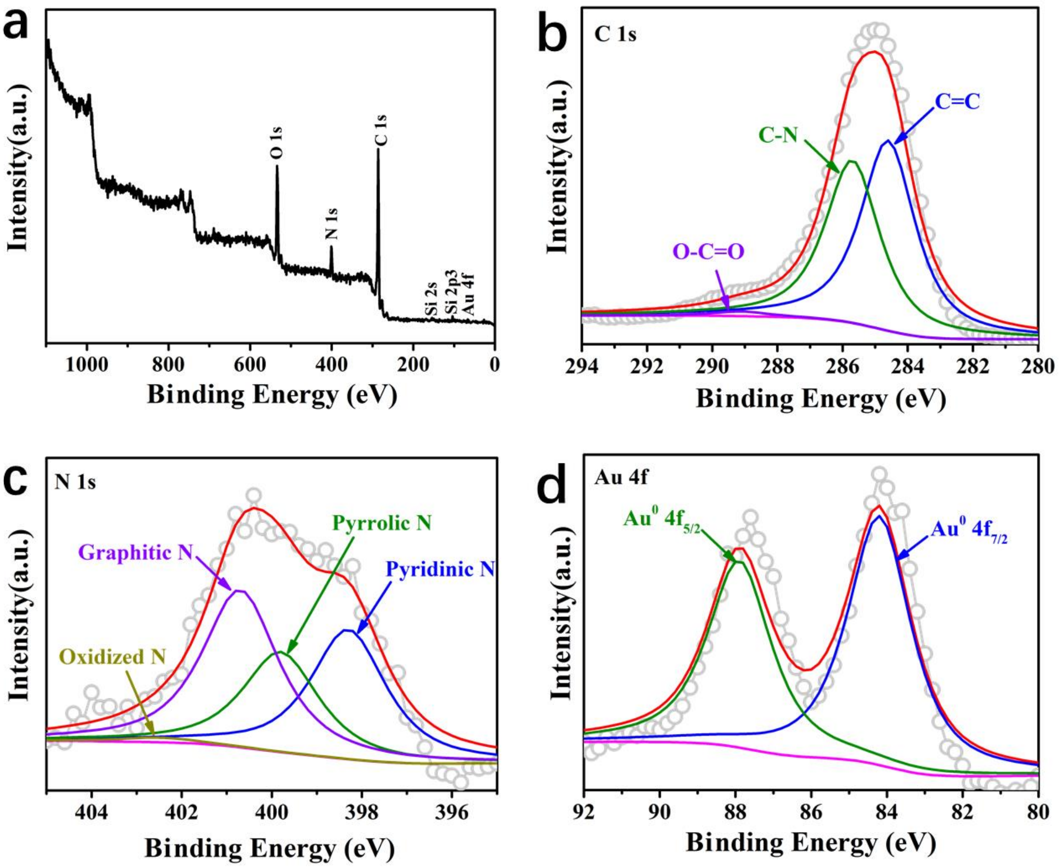

Figure S13. XPS survey spectrum (a) and high resolution spectra of C 1s (b), N 1s (c) and Au 4f (d) for Au@MMC-V microspheres.

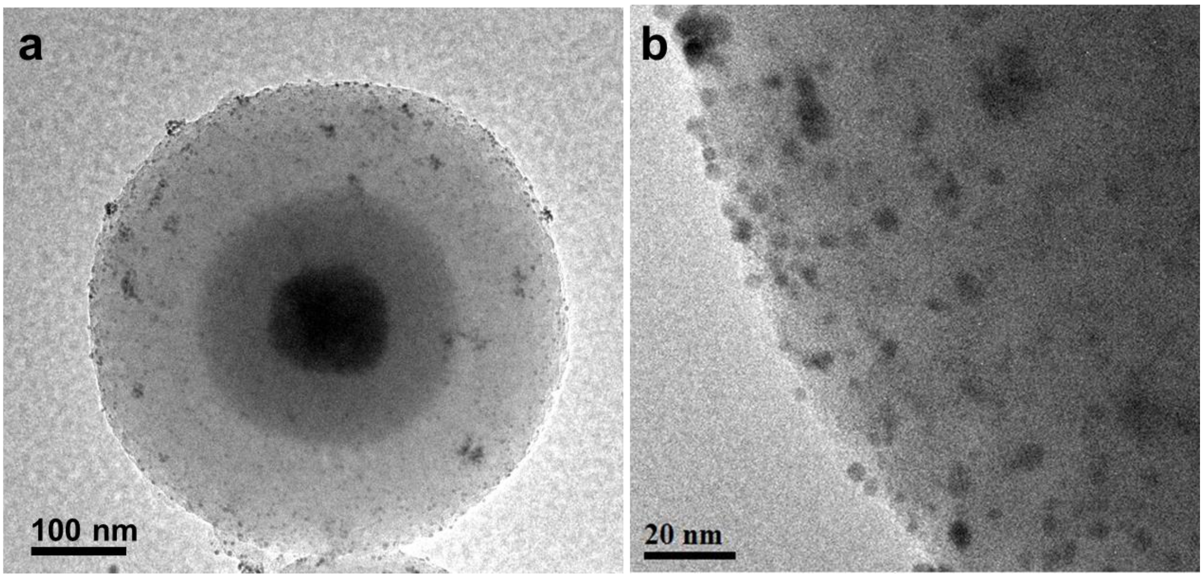

Figure S14. TEM images of Au@MMC-V microspheres recycled after running catalysis for 6 runs.
